# Between Light and Shading: Morphological, Biochemical, and Metabolomics Insights Into the Influence of Blue Photoselective Shading on Vegetable Seedlings

**DOI:** 10.3389/fpls.2022.890830

**Published:** 2022-05-25

**Authors:** Luigi Formisano, Begoña Miras-Moreno, Michele Ciriello, Leilei Zhang, Stefania De Pascale, Luigi Lucini, Youssef Rouphael

**Affiliations:** ^1^Department of Agricultural Sciences, University of Naples Federico II, Portici, Italy; ^2^Department for Sustainable Food Process, DiSTAS, Università Cattolica del Sacro Cuore, Piacenza, Italy

**Keywords:** shading screen, plantlets, sturdiness index, red:blue ratio, metabolomics, plant metabolism, lipid biosynthesis, phenylpropanoids

## Abstract

High nursery densities reduce the seedling quality due to the competition for light. High light intensity, shading, and blue light depletion activate morphophysiological and metabolomic responses in plants, resulting in size modification to gain an advantage over neighboring plants. Our research aimed to unravel the effects of light intensity and quality on nursery seedlings at the morphological and biochemical levels. To this aim, the effect of black shading and blue photoselective shading nets were investigated in terms of morphometric, ionomic, and untargeted metabolomics signatures in *Cucurbita pepo* L., *Citrullus lanatus* L., *Solanum lycopersicum* L., and *Solanum melongena* L. seedlings. Plant height, diameter, sturdiness index, leaf area, specific leaf area, shoot/root ratio, and mineral content (by ion chromatography-IC) were evaluated. In *C. pepo* L and *C. lanatus* L., the blue net reduced the shoot/root and chlorophyll a/b ratios and increased stem diameter and total chlorophyll content. The black net increased plant height, stem diameter, and sturdiness index in *Solanum lycopersicum* L. and *Solanum melongena* L. At the same time, unshading conditions reduced leaf area, specific leaf area, shoot/root ratio, and total chlorophyll content. The blue net improved the sturdiness index and quality of *C. pepo* L. and *C. lanatus* L. Such impact on morphological parameters induced by the different shading conditions was corroborated by a significant modulation at the metabolomics level. Untargeted metabolomic phytochemical signatures of the selected plants, and the subsequent multivariate analysis coupled to pathway analysis, allowed highlighting a broad and diverse biochemical modulation. Metabolomics revealed that both primary and secondary metabolism were largely affected by the different shading conditions, regardless of the species considered. A common pattern arose to point at the activation of plant energy metabolism and lipid biosynthesis, together with a generalized down accumulation of several secondary metabolites, particularly phenylpropanoids. Our findings indicate an intriguing scientific interest in the effects of selective shading and its application to other species and different phenological stages.

## Introduction

Nursery activities are the “backbone” of modern agricultural production systems, in addition to asserting additional assets for social and economic sustainability (Timpanaro et al., 2018; Rani et al., 2019; Rouphael et al., 2021). In the agricultural scene, the horticultural and nursery sectors have grown over the years due to their distinctive dynamism, the ongoing technological upgrading, and investment in new growing techniques to meet the increasing demand for high-quality seedlings (i.e., healthy, vigorous, and balanced development) and adaptability to different climates and soils. The adaptability of seedlings to changing environmental conditions is the cornerstone of nursery production. Drought, soil salinity, temperature, humidity, and sub-optimal nutrient levels are examples of environmental pressures that imperil seedling establishment, performance, and survival in their natural habitats (Franco et al., 2006). Pre-conditioning nursery techniques are crucial to producing robust plants with adequate morphology and high levels of organic reserves. These latter attributes are critical to ensure increased vegetative vigor during seedling establishment (Franco et al., 2006). Direct morphological parameters (such as plant height, stem diameter, root length, dry weight, and leaf area), derived parameters (such as the sturdiness index, the shoot/root ratio, and the leaf area ratio), and physiological parameters (such as mineral and chlorophyll content) are usually used for seedling quality assessment (Lin et al., 2019). For example, a lower sturdiness index (i.e., the ratio of stem height to stem diameter) reduces seedling lodging, while a low shoot/root ratio reduces mortality rates when grown in drought environments (Franco et al., 2006; Mañas et al., 2009). Mañas et al. (2009) reported that higher shoot dry weight (high content of photosynthetic reserves) increased the vigor and survival of seedlings after transplanting. At the same time, Grossnickle (2005) pointed out that high leaf area (excessive shoot growth) could lead to severe transplant shock as a consequence of water imbalances between shoot and root. Finally, a thicker stem and a larger root system increased resistance to transplant shock (Grossnickle and MacDonald, 2018b).

In plants, vegetative growth and development depend on cell division, cell elongation, directional growth, and branching (Ouzounis et al., 2015), where light is one of the environmental parameters that can drive many of these processes (Devlin et al., 2007). Plants are light-dependent and therefore have evolved sophisticated photoreceptors that control specific biochemical and physiological aspects to maximize photosynthetic performance by adapting to a specific light environment (Folta and Carvalho, 2015; Paradiso and Proietti, 2021). Usually, light-demanding species have higher photosynthetic activity, thicker roots, and long shoots. In contrast, shade-tolerating species increase leaf size under shading, show a higher chlorophyll content, and decrease their light compensation point to balance the reduced photosynthetic activity (Walter and Nagel, 2006; Ferrante and Mariani, 2018; Poorter et al., 2019).

However, modern nursery techniques based on high planting density can reduce seedling quality due to unwanted changes in key morphological parameters (Wang X. et al., 2020). In high-density seedlings, tight spaces cause a strenuous struggle for light, a scenario that triggers photo-morphogenetic adaptations to increase competitiveness among plants (Keuskamp et al., 2012; Paradiso and Proietti, 2021). To manage the challenging relationship between neighbors, plants can rely on two strategies: react (avoidance) or adapt (tolerance) (Morelli and Ruberti, 2002). Plants, through photoreceptors, detect shading as a reduced intensity in and/or changes in light quality (Morelli and Ruberti, 2002; Keuskamp et al., 2012). For example, depletion of blue radiation is an indicator of effective shading to which plants respond by elongating the stem and increasing the angle of incidence of the leaves (hyponastia) to take advantage of neighboring plants (Keuskamp et al., 2012). Stimulus-response induction is mediated by cryptochrome, a phototropin photoreceptor involved in the uptake of blue light and contributes largely to plant shape (Folta and Carvalho, 2015). Blue light depletion caused by self-shading can result in excessive stem and shoot growth, an undesirable aspect for nursery seedlings. Thus, forcing producers to use chemical size regulators that inhibit gibberellin production, resulting in shorter internodes and more controlled plant growth. Although morphological responses to blue light are genotype-dependent and can differ even among genotypes (Huché-Thélier et al., 2016), increasing blue radiation through alternative non-chemical methods could be a viable and environmentally sustainable aid to reduce nursery seedling size. However, the application of the blue spectrum in seedling cultivation has rarely been studied or documented in scientific manuscripts, and very little is known about the metabolic changes associated with planting exposition to the blue net. Considering the direct linkage between light and essential processes (not limited to photosynthesis) and its connection to the carbon and nitrogen fluxes (Li et al., 2020), studying the metabolic processes underlying selective shading is crucial in understanding the profound impact of shading in crops. In this sense, the hypothesis-free comprehensive profiling provided by untargeted metabolomics may provide a holistic overview of the different biochemical processes triggered by selective shading, thus providing valuable insights into the metabolic reprogramming induced in crops.

Based on these assumptions, the objective of our research was to evaluate the effects of intensity (No shading) and quality (Blue net) of light on morphometric and quality parameters, colorimetric indices, mineral concentration, and pigment concentration, in *Solanaceae* and *Cucurbitaceae* seedlings, compared to ordinary summer shading practices in the nursery (Black net). At the same time, metabolic reprogramming was investigated through untargeted metabolomics in light-demanding species such as zucchini squash (*Cucurbita pepo* L.), watermelon (*Citrullus lanatus* L.), tomato (*Solanum lycopersicum* L.), and eggplant (*Solanum melongena* L.) seedlings for nursery production in the Mediterranean environment. To our knowledge, this is the first study that has investigated these aspects and will be of prime interest to seedling producers.

## Materials and Methods

### Experimental Design, Plant Material, and Technical Characteristics of the Nets

The experimental trial evaluated the intensity and quality of light on nursery seedlings. It was carried out in spring-summer 2021 at “Vivai Giuseppe Bene” nursery farm, located in Poggiomarino (Naples, Italy, 40°79′ N, 14°53′ E, 46 m.s.l.). The experiment protocol was based on comparing a blue photoselective shading net, a commercial black shading net (as of Control), and a transparent plastic film in ethyl vinyl acetate that covered high tunnels 10 m wide, 35 m long, and 3.5 and 5 m high at the eaves and ridge, respectively. The shading nets were in factorial combination with *Cucurbitaceae* shading-demanding species seedlings as zucchini squash (*Cucurbita pepo* L. cv. San Pasquale, Pagano Domenico and Figli, Scafati, Italy) and watermelon (*Citrullus lanatus* L. cv. Crimson Sweet, Pagano Domenico and Figli, Scafati, Italy) and *Solanaceae* shading-demanding species seedlings as tomato (*Solanum lycopersicum* L., cv. OR Grandborghese, Four-Blumen Vegetable seeds, Piacenza, Italy) and eggplant (*Solanum melongena* L., cv. Mirabelle F1—Seminis, Milan, Italy), sown in polystyrene plug trays (experimental unit) (*Cucurbitaceae*: 60 plants/tray; *Solanaceae*: 180 plants/tray). The experimental design was randomized into three replicates. Seeds were sown on June 29, 2021, covered with a thin layer of vermiculite and placed in a germination chamber for 36 h (until seed coats cracked and the shoots just started to emerge). On July 2, the trays were moved under the nets. The characteristics of the nets were as follows: (1) ChromatiNet^§^ Blue (hereafter “Blue net”; shading factor: 40%; red:blue ratio = 1; Ginegar Plastic Products Ltd., Kibbutz Ginegar, Israel); (2) 2635NE Agri LDF black (hereafter “Black net”; shading factor: 40%; red:blue ratio = 1.4; Arrigoni S.p.A, Uggiate Trevano, Italy); and (3) Sunlux 200 EVO plastic film (hereinafter “No shading”; shading factor: 20%; red:blue ratio = 1.4; Comagri S.r.l., Grumello del Monte, Italy). The photosynthetically active radiation (PAR) was continuously recorded using WatchDog A150 dataloggers (Spectrum Technologies Inc., Aurora, IL, United States) (Supplementary Figure 1) while the red:blue ratio, the spectral irradiance (W m^–2^ nm^–1^), and the degree of light extinction of the nets were evaluated using a portable spectral radiometer (MSC15, Gigahertz-Optik, Turkenfeld, Germany) (Supplementary Figures 2, 3).

### Sampling and Determination of Morphometric and Quality Indices of Seedlings

Seedlings were sampled when they reached their marketable size (at two true leaves for zucchini squash and watermelon and three true leaves for tomato and eggplant). Specifically, zucchini squash, watermelon, tomato, and eggplant seedlings were sampled at 14, 19, 21, and 27 days after sowing, respectively. Twenty defect-free plants per experimental unit were harvested (avoiding border plants), weighed, and separated into leaves, stems, and roots. Plant height (cm plant^–1^) was measured, and leaf area (cm^2^ plant^–1^) was assessed by digital image analysis using ImageJ v1.52a software (United States National Institutes of Health, Bethesda, MD, United States). A leaf tissue subsample was immediately stored at –20°C for pigment determination, while another subsample was immediately frozen at –80°C and subjected to a freeze-drying cycle (Alpha 1–4 Martin Christ Gefriertrocknungsanlagen GmbH, Osterode am Harz, Germany) for metabolomic analyzes. The diameter of the stem was measured using a digital caliper (± 0.02 mm accuracy; RS PRO, Sesto San Giovanni, Italy). The roots were gently cleaned in water, spread on a graph paper, and measured in length (cm plant^–1^). All tissues collected were oven-dried at 70°C to constant weight (∼72 h) to determine the dry weight (mg plant^–1^). The dried leaves and stems were ground with an MF10.1 cutting head mill (IKA^®^, Staufen im Breisgau, Germany) and sieved with an MF0.5 sieve (hole size 0.5 mm; IKA^®^, Staufen im Breisgau, Germany) for mineral determination. Then, derived quality indices such as the shoot/root ratio, sturdiness index (stem height/root collar diameter), and specific leaf area (LAR, cm^2^ mg^–1^ plant^–1^; leaf area/total dry weight) were calculated.

### Soil Plant Analysis Development Index and Leaf Color Determination

At harvest, the soil plant analysis development (SPAD) index (greenness index) was measured on twenty young and fully expanded leaves of each experimental unit using a portable chlorophyll meter (SPAD-502, Minolta Camera Co., Ltd., Osaka, Japan) and CIELab colorimetric coordinates by a Minolta CR-300 colorimeter (Minolta Co., Ltd., Osaka, Japan) calibrated with a corresponding Minolta standard.

### Mineral Determination

The determination of cations [potassium (K), calcium (Ca), and magnesium (Mg)] and anions [nitrate and phosphorus (P)] in zucchini squash, watermelon, tomato, and eggplant seedlings were assessed by ion chromatography according to the method described in detail by Formisano et al. (2021a). Briefly, 0.25 g of finely ground dry material was mixed with 50 ml of ultrapure water (Arium^®^ Advance EDI pure water system, Sartorius, Goettingen, Germany), placed in a shaking water bath for 10 min (100 rpm; Julabo, Seelbach, Baden-Württemberg, Germany), and centrifuged for 10 min (6,000 rpm, R-10M centrifuges, Remi Elektrotechnik Ltd., Mumbai, India). A 0.25-μL aliquot of the supernatant was filtered and processed by anionic chromatography coupled to an electrical conductivity detector (ICS-3000, Thermo Scientific™ Dionex™, Sunnyvale, CA, United States). Columns, pre-columns, and self-regenerating suppressors were purchased from Thermo Scientific™ Dionex™ (Sunnyvale, CA, United States). Cations separation was performed isocratically using 25 mM methanesulfonic acid as an eluent (Sigma Aldrich, Milan, Italy). Anions separation was performed in a gradient mode (5–30 mM KOH with a 1.5 mL min^–1^ flow). The integration and quantification of minerals were performed using Chromeleon™ 6.8 Chromatography Data System (CDS) software (Thermo Scientific™ Dionex™, Sunnyvale, CA, United States), comparing the peak areas of the samples with those of the standards. Anions and cations concentrations were expressed as g kg^–1^ dry weight (dw), except for nitrate, which was expressed as mg kg^–1^ fresh weight (fw). Each treatment was analyzed in triplicate.

### Pigments Determination

Pigments (total chlorophyll, a, b, and carotenoids) were determined as described by Formisano et al. (2021b). Briefly, 0.5 g of fresh leaves were extracted in ammonia acetone, crushed in a ceramic mortar, and centrifuged at 2,000 rpm for 10 min using an R-10 M centrifuge (Remi Elektrotechnik Limited, Mumbai, India). The contents of chlorophyll a, chlorophyll b, and carotenoids were determined by UV-Vis spectrophotometry (ONDA V-10 Plus, Giorgio Bormac srl, Carpi, Italy) with an absorbance of 647, 664, and 470 nm, respectively. Total chlorophylls were calculated as chlorophyll a + chlorophyll b. In addition, the chlorophyll a/chlorophyll b ratio was calculated. Total chlorophylls and carotenoids were expressed as mg g^–1^ fw.

### Metabolomics Analysis

The untargeted metabolomics profiling of the four seedling species was carried out by extracting 0.5 g of dried leaves in 5 mL of extraction solvents, composed of 80% *v/v* methanol + 20% *v/v* ultrapure water and acidified with 0.1% formic acid (Merck KGaA, Darmstadt, Germany). The samples were subsequently homogenized using a Polytron^®^ PT1200 E (Kinematica AG, Malters, Switzerland) homogenizer and centrifuged at 8,000 × *g* for 15 min. The supernatants were filtered with a 0.22 mm syringe filter and transferred in glass vials ready to be injected (volume of 6 μL) into the ultra-high-pressure liquid chromatography coupled to a quadrupole time of flight mass spectrometer (UHPLC-QTOF-MS; Agilent Technologies, Stevens Creek Blvd, Santa Clara, CA, United States) as previously reported (Benjamin et al., 2019). In detail, the chromatographic separation was achieved by using an Agilent InfinityLab Poroshell 120 pentafluorophenyl (PFP) column (2.1 × 100 mm, 1.9 μm) (Agilent Technologies, Stevens Creek Blvd, Santa Clara, CA, United States) and a binary mixture of water and acetonitrile acidified with 0.1% (*v/v*) formic acid as mobile phase (LC-MS grade, VWR, Milan, Italy). The data analysis after the samples acquisition was carried out using Agilent Profinder B 0.10.0 (Agilent Technologies, Stevens Creek Blvd, Santa Clara, CA, United States) in order to align and annotate the features according to the “find-by-formula” algorithm against the PlantCyc 12.6 database (Schläpfer et al., 2017), retaining only those compounds putatively annotated within 75% of replications in at least one condition (Lucini et al., 2019). Monoisotopic accurate mass was used together with the entire isotopic profile, achieving level 2 of confidence in annotation (Salek et al., 2015).

### Statistical Analysis

Data from each species were subjected to a one-way ANOVA using IBM SPSS Statistics software (SPSS Inc., Chicago, IL, United States) version 26 for Windows 11 and presented as mean ± standard error, *n* = 3. Statistical significance was determined using Tukey’s HSD test at the *p* = 0.05 level. All seedling responses to changing light intensity and quality on morphometric and quality indices, minerals, colorimetric parameters, and pigment accumulation were summarized *via* color heatmaps generated using the web-based tool ClustVis^1^. The Euclidean distance was used as a measure of similarity and hierarchical clustering with full link heatmaps, and the data were normalized [ln(x + 1)] and displayed using a false-color scale (red = increase in values; blue = decrease in values) (Modarelli et al., 2020).

The chemometric interpretation of the metabolic features was conducted with Mass Profiler Professional B 0.15.1 (Agilent Technologies, Stevens Creek Blvd, Santa Clara, CA, United States), as previously described in our study (Benjamin et al., 2019). Using this software, the raw metabolomic data set was transformed and normalized and then used for fold-change analysis. For this purpose, supervised orthogonal projections to latent structures discriminant analysis (OPLS-DA), using SIMCA 16 (Umetrics, Malmo, Sweden), was performed considering all the species together and only the nets as a factor. Subsequently, the OPLS-DA model was validated, and model fitness parameters (goodness of fit: R^2^Y; goodness of prediction: Q^2^Y) were inspired through the permutation test (*n* = 100) and Hotelling’s T2 (95% and 99% confidence limit for the suspect and strong outliers, respectively). Then, the variable importance in projection (VIP ≥ 1.3) was adopted to identify discriminant metabolites among different treatments for the four species, and the resulted compounds were subjected to a fold-change (FC) to better understand the differences among treatments compared to the unshading plants. After that, VIP markers were uploaded into the Omic Viewer Pathway Tool of PlantCyc (Stanford, CA, United States) to identify the pathways and processes affected by treatments.

## Results and Discussion

### Effects of Light Intensity and Quality on Morphometric and Seedling Quality Indices

Light plays a pivotal role in regulating physiological and critical processes in plants (Devlin et al., 2007; Folta and Carvalho, 2015; Ajdanian et al., 2019). Through complex mechanisms, plants capture light reaching their leaves and activate molecular pathways to acclimate to specific light environments (Paik and Huq, 2019). However, the productive performance also depends on light quality, which can trigger particular gene expressions that have a different impact on plant survival (Franco et al., 2006; Huché-Thélier et al., 2016; Lin et al., 2019; Paradiso and Proietti, 2021). The morphometric indices in Table 1 show a significant effect of light intensity and quality on plant height. Except for zucchini squash seedlings, shading treatments (Black and Blue net) increased, on average, watermelon, tomato, and eggplant seedling size by 24.36%, 35.91%, and 28.04%, respectively, compared to the unshaded treatment (No shading).

**TABLE 1:** Effects of shading and light quality on morphometric indices of zucchini squash (*Cucurbita pepo* L.), watermelon (*Citrullus lanatus L.)*, tomato (*Solanum lycopersicum L.)*, and eggplant (*Solanum melongena L.)* seedlings.

Crop	Treatment	Plant height	Leaf area	Shoot dry weight	Root dry weight	Shoot/root ratio
		(cm plant^–1^)	(cm^2^ plant^–1^)	(mg plant^–1^)		
	No shading	2.364 ± 0.018*b*	43.675 ± 0.330*a*	356.167 ± 9.076*a*	108.433 ± 3.795	3.674 ± 0.352
*Zucchini squash*	Black net	2.821 ± 0.014*a*	43.477 ± 0.135*a*	340.233 ± 2.747*a*	100.067 ± 1.486	3.811 ± 0.007
	Blue net	2.338 ± 0.019*b*	42.254 ± 0.147*b*	304.300 ± 7.199*b*	101.433 ± 0.837	3.458 ± 0.024
	Significance	***	**	**	*Ns*	*ns*
	No shading	2.820 ± 0.091*c*	20.561 ± 0.690*b*	321.867 ± 1.068*a*	86.200 ± 1.914*b*	4.201 ± 0.117*a*
*Watermelon*	Black net	3.752 ± 0.087*a*	21.904 ± 0.609*b*	296.100 ± 5.575*b*	77.100 ± 1.415*c*	3.980 ± 0.052*a*
	Blue net	3.262 ± 0.045*b*	24.893 ± 0.323*a*	327.033 ± 1.281*a*	100.567 ± 0.437*a*	3.404 ± 0.055*b*
	Significance	***	**	***	***	***
	No shading	5.309 ± 0.123*c*	7.421 ± 0.069*c*	119.133 ± 2.811*b*	44.067 ± 0.606*b*	2.694 ± 0.023*b*
*Tomato*	Black net	7.915 ± 0.077*a*	11.776 ± 0.053*a*	148.933 ± 6.438*a*	52.933 ± 1.386*a*	2.820 ± 0.059*ab*
	Blue net	6.516 ± 0.095*b*	10.568 ± 0.030*b*	133.267 ± 3.689*ab*	44.900 ± 0.361*b*	2.962 ± 0.055*a*
	Significance	***	***	*	***	*
	No shading	3.589 ± 0.011*c*	17.587 ± 0.613*c*	127.833 ± 2.210*b*	71.567 ± 0.940*a*	1.807 ± 0.073*c*
*Eggplant*	Black net	5.046 ± 0.086*a*	27.274 ± 0.504*b*	157.233 ± 3.830*a*	61.100 ± 1.206*b*	2.985 ± 0.119*a*
	Blue net	4.145 ± 0.031*b*	31.425 ± 0.335*a*	163.900 ± 2.743*a*	64.700 ± 0.351*b*	2.622 ± 0.033*b*
	Significance	***	***	***	***	***

Different letters within columns indicate significant mean differences according to Tukey’s HSD test (p = 0.05). ns, *, ^**^ and ^***^ denote non-significant or significant effects at p ≤ 0.05, 0.01, and 0.001, respectively. Data are mean values ± standard error, n = 3.

**FIGURE 1:**
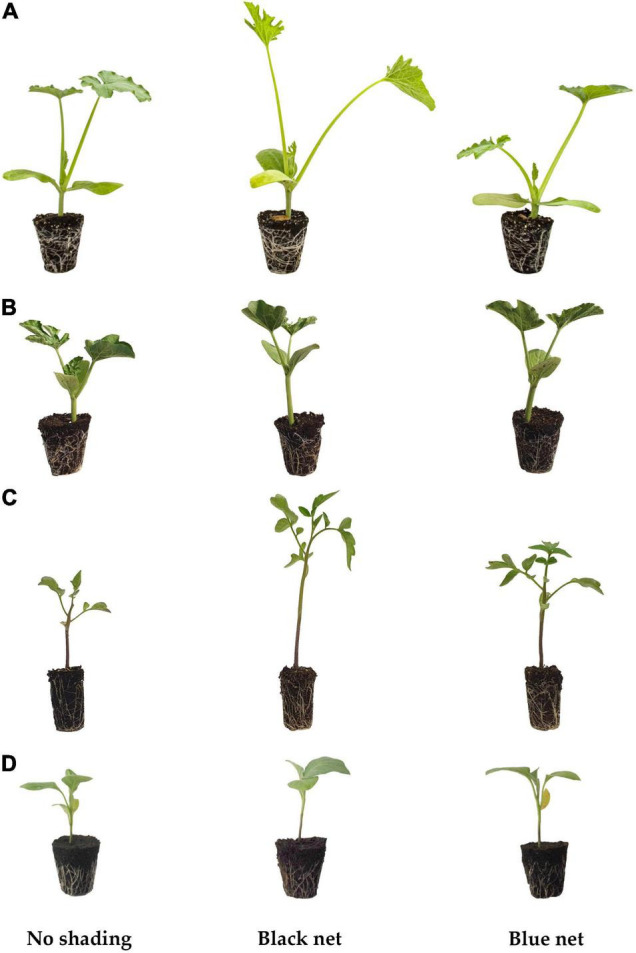
Illustrative picture of the effects of light intensity and quality on the seedling height of zucchini squash (*Cucurbita pepo* L.) **(A)**, watermelon (*Citrullus lanatus* L.) **(B)**, tomato (*Solanum lycopersicum* L.) **(C)**, and eggplant (*Solanum melongena* L.) **(D)**.

The increase in plant height in our experiment is a typical phenotypic response to the so-called “shade avoidance syndrome” in light-demanding plants (Figure 1; Keuskamp et al., 2012). In the shade, light-demanding plants detect light depletion through specific photoreceptors such as phytochromes (Morelli and Ruberti, 2002), and they trigger morphological changes that promote stem elongation through a complex network of hormones and transcriptional regulators (Smith, 1995; Smith and Whitelam, 1997; Ballaré, 1999; Morelli and Ruberti, 2000). As reported by Casal (2013) and Ballaré and Pierik (2017), under shading, the active state of phytochrome B (Pfr) is converted to the inactive state (Pr). This conversion releases the negative feedback of phytochrome B on phytochrome interacting factors (PIFs), leading to auxin and gibberellin production that results in cell elongation, thus ensuring better light accessibility to plants. Similarly, plants’ changes in the spectral light quality are detected as a warning signal of future competition. The literature has well documented that the depletion of blue light, or its limited availability, can prompt stem elongation due to an attenuation of the cryptochrome-PIFs interaction (Folta and Carvalho, 2015; Ma et al., 2016; Pedmale et al., 2016). In the present investigation, increasing the percentage of blue light in the light spectrum by photoselective blue net (Black net: R/B = 1.4, Blue net: R/B = 1; Supplementary Figure 2) decreased the height of plants, compared to the black net (Table 1). Similar to phytochromes, the effects of blue light on cryptochromes generate signals that suppress gibberellin and auxin syntheses, affecting gene expression involved in elongation repression (Folta et al., 2003). Our results are consistent with previous studies on light-demanding plants such as tomato seedlings (Glowacka, 2004; Nanya et al., 2012; Wollaeger and Runkle, 2014; Hernández et al., 2016; Snowden et al., 2016), cucumber (*Cucumis sativus* L.) (Snowden, 2015; Hernández and Kubota, 2016), broccoli (*Brassica oleracea* var. italica), kohlrabi (*Brassica oleracea* Gongylodes) (Li et al., 2019) and pepper (*Capsicum annuum* L.) (Snowden, 2015) seedlings grown under an LED light.

The leaf area showed divergent trends between *Cucurbitaceae* and *Solanaceae* (Table 1). The unshading condition reduced leaf area in tomato and eggplant seedlings compared to the shading treatments. Probably, under high light intensity, light-demanding plants decrease leaf expansion to catch less light and limit any damage to the photosystem. The reduction in leaf area also explains the lower shoot dry weight registered for the same species (Table 1). Shoot dry weight reflects the net gain from photosynthesis, and its accumulation is mainly driven by the source: the sink of photosynthesis. In fact, high shoot dry weight indicates a better growth potential (Mañas et al., 2009). However, Grossnickle (2005) suggested that a high leaf weight could lead to increased transplant stress under suboptimal conditions (e.g., drought and heat) because the root system might not provide sufficient water to the leaves to maintain adequate water balance during the establishment phase. The different responses observed for leaf area and shoot dry weight in *Cucurbitaceae* could be derived from their less permanence in the nursery (12–15 days for *Cucurbitaceae* vs. 20–30 days for *Solanaceae*) and the genotypic effect (Table 1). In zucchini squash, regardless of light intensity, the ratio R/B = 1.4 (No shading and black net) increased leaf area and shoot dry weight, which is consistent with the findings of Hernández and Kubota (2014), who reported an increase in shoot dry weight due to a higher allocation of dry weight to the leaves. In contrast, as in eggplant seedlings, the highest leaf area in watermelon was obtained under the blue net (R/B = 1). Our results are in agreement with the reviewed literature, where XiaoYing et al. (2011) reported that an R/B = 1 ratio promoted leaf expansion in tomato seedlings by improving light absorption, while Lian et al. (2002) and Kim et al. (2004) reported similar results on light-demanding species such as Lilium (*Lilium oriental* “Pesaro”) and Chrysanthemum (*Dendranthema grandiflorum* Kitam “Cheonsu”).

Except for zucchini squash seedlings, shading increased the leaf area ratio (LAR; Table 2). As Freschet et al. (2018) reported, the LAR increased under shading due to the increased leaf area rather than the dry weight of the leaf. This result is confirmed in tomato and eggplant seedlings, where leaf areas were, on average, 50.54 and 66.88% higher than that in the unshading condition.

**TABLE 2:** Effects of shading and light quality on quality indices of zucchini squash (*C. pepo*), watermelon (*C. lanatus)*, tomato (*S. lycopersicum)*, and eggplant (*S. melongena)* seedlings.

Crop	Treatment	Stem diameter	Root length	Sturdiness index	Leaf area ratio
		(cm plant^–1^)			(cm^2^ mg^–1^ plant^–1^)
	No shading	0.442 ± 0.001ab	11.042 ± 0.081c	5.416 ± 0.022b	0.123 ± 0.002*b*
*Zucchini squash*	Black net	0.436 ± 0.003b	12.077 ± 0.100a	6.682 ± 0.410a	0.128 ± 0.001b
	Blue net	0.456 ± 0.007a	11.519 ± 0.029b	5.249 ± 0.047b	0.139 ± 0.003a
	Significance	*	***	**	**
	No shading	0.433 ± 0.003b	11.043 ± 0.536b	6.267 ± 0.134c	0.064 ± 0.002b
*Watermelon*	Black net	0.404 ± 0.006c	12.995 ± 0.173a	9.974 ± 0.189a	0.074 ± 0.001a
	Blue net	0.477 ± 0.002a	11.633 ± 0.394ab	7.175 ± 0.159b	0.076 ± 0.001a
	Significance	***	*	***	**
	No shading	0.262 ± 0.003	11.331 ± 0.176a	20.304 ± 0.927c	0.062 ± 0.001b
*Tomato*	Black net	0.266 ± 0.004	10.876 ± 0.426a	30.508 ± 0.854a	0.079 ± 0.003a
	Blue net	0.261 ± 0.000	9.371 ± 0.173b	26.993 ± 0.043b	0.079 ± 0.002a
	Significance	*ns*	**	***	**
	No shading	0.232 ± 0.006b	10.048 ± 0.075b	15.600 ± 0.170b	0.137 ± 0.003b
*Eggplant*	Black net	0.250 ± 0.003a	10.929 ± 0.137a	20.312 ± 0.115a	0.174 ± 0.007a
	Blue net	0.261 ± 0.002a	11.314 ± 0.187a	15.914 ± 0.155b	0.192 ± 0.003a
	Significance	**	**	***	***

Different letters within columns indicate significant mean differences according to Tukey’s HSD test (p = 0.05). ns, *** and *** denote non-significant or significant effects at p ≤ 0.05, 0.01, and 0.001, respectively. Data are mean values ± standard error, n = 3.

Regarding the effects of light quality on the root system, it should be noted that blue light promoted root growth in watermelon seedlings, resulting in a lower shoot/root ratio (Table 1). The shoot/root ratio is a crucial index for seedlings as it correlates with their survival (Franco et al., 2006). Indeed, reducing the shoot/root ratio reduces the plant mortality rate at transplant establishments (Franco et al., 2006). An inadequately developed root system cannot provide enough water to large shoots, making plants unsuitable for active growth (Johkan et al., 2010). In zucchini squash seedlings, the intensity and quality of light did not affect root dry weight and, consequently, shoot/root ratio. While in shading treatments, root length increased, on average, by 6.85%, compared to the unshading condition (Table 2). A different situation was observed for *Solanaceae*. In tomato seedlings, the black net promoted root growth (> root dry weight), while the same trend was not found in eggplant seedlings, where the blue net lowered the shoot/root ratio in shading conditions (Table 1). However, the lowest shoot/root ratio (1.807) was recorded under unshading conditions due to the higher root dry weight (Table 1).

In addition to the root system and plant height, the diameter of the stem plays a crucial role in seedling survival and growth. A larger stem diameter reduces transplant stress by improving water transport and uptake (Grossnickle and MacDonald, 2018a,b). Compared to the black net, the blue net increased the stem diameter in zucchini squash and watermelon, while no effect was observed in tomato seedlings (Table 2). The lowest value was obtained in the No shading treatment in eggplant seedlings, which justified the lower shoot dry weight (Tables 1, 2). As Grossnickle and MacDonald (2018b) indicated, the divergent results revealed that the relationship between big stem diameter and seedling survival is not universal. The effects of blue light on stem diameter increase were previously reported in mature light-demanding plants of canola (*Brassica napus* “Modena”) (Tehrani et al., 2016) and cress (*Lepidium sativum* L.) (Ajdanian et al., 2019) grown under the LED light.

The different responses of plants to the quantity and quality of light on the height and diameter of the stem were mirrored in the sturdiness index (Table 2). In nursery production, a lower sturdiness index indicates a better-quality plant and is an indirect parameter for evaluating the seedlings’ survival rate and growth performance (Grossnickle and MacDonald, 2018b). In our study, regardless of family and species, unshading conditions and the blue net increased plant compactness (lower sturdiness index) compared to the black net (Table 2). The increased plant compactness was directly related to the plant height reduction and stem diameter increase obtained in the above treatments (Tables 1, 2).

### Effects of Light Intensity and Quality on Colorimetric Indices of Seedlings

The perception of the world around us is determined by the mutual interaction between physical stimuli and sensory responses. Color is one of the most important sensory attributes, influencing consumer choice and decision and predicting sensorial quality attributes in food (Pathare et al., 2013). However, perceived color differences in plants are due to the concentration of natural pigments such as carotenoids, chlorophylls, anthocyanins, and flavonoids that differ according to several factors such as genotype, phenological stage, postharvest treatments, and especially growth conditions (Pathare et al., 2013). Although there are no scientific contributions in the literature highlighting the key role of non-edible plant color in consumer preferences, color is also crucial in the nursery production of premium quality seedlings characterized by high compactness and vivid colors. Glossy, bright green leaf surfaces indicate high quality, associated with good water and nutritional status, and a good, well-formed, non-spiralized, non-senescent root system. Except for zucchini squash, the findings in Table 3 showed a significant influence of the different treatments on the CIELab colorimetric parameters and the SPAD index. However, the species’ response to the change in the intensity and quality of light was not univocal. In watermelon, the effects of the blue net on the morphometric and qualitative parameters were coupled with an increase in the SPAD index and a reduction in b*, compared to the other treatments (black net and No shading; Tables 1, 2). However, the blue net led to the lowest L* (46.475) while the highest L* (47.459) was obtained in the No shading treatment. The same increasing trend was observed for L* and b* in zucchini squash in the No shading treatment (Table 3). In *Solanaceae*, the highest SPAD index was obtained in the No shading treatment. However, this finding was not associated with improved morphometric and qualitative indexes of plants grown under the same conditions (Tables 1, 2). However, the most negative a* values were recorded under the black net. The lowest L* in tomato (47.749) was recorded in the No shading treatment, while in eggplant (43.879), it was recorded in the blue net treatment.

**TABLE 3:** Effects of shading and light quality on SPAD index and CIELab colorimetric parameters of zucchini squash (*C. pepo*), watermelon (*C. lanatus)*, tomato (*S. lycopersicum)*, and eggplant (*S. melongena)* seedlings.

Crop	Treatment	SPAD index	L*	a*	b*
	No shading	41.102 ± 0.564	45.861 ± 0.308a	−16.977 ± 0.272	24.034 ± 0.346a
*Zucchini squash*	Black net	41.593 ± 0.483	42.325 ± 0.368b	−16.017 ± 0.126	21.625 ± 0.189b
	Blue net	42.212 ± 0.054	41.576 ± 0.380b	−16.248 ± 0.366	21.469 ± 0.255b

	Significance	ns	***	ns	***

	No shading	47.733 ± 0.044b	47.459 ± 0.056a	−15.696 ± 0.056b	24.374 ± 0.107a
*Watermelon*	Black net	47.421 ± 0.061b	46.935 ± 0.134b	−14.850 ± 0.104a	21.779 ± 0.146b
	Blue net	48.932 ± 0.128a	46.475 ± 0.092c	−14.797 ± 0.023a	21.245 ± 0.018c

	Significance	***	***	***	***

	No shading	43.369 ± 0.477a	47.749 ± 0.202b	−15.596 ± 0.085a	25.400 ± 0.211b
*Tomato*	Black net	37.055 ± 0.486b	49.749 ± 0.172a	−17.784 ± 0.035c	30.038 ± 0.080a
	Blue net	37.157 ± 0.127b	49.943 ± 0.061a	−17.405 ± 0.059b	29.895 ± 0.044a

	Significance	***	***	***	***

	No shading	39.955 ± 0.068a	44.750 ± 0.236b	−13.294 ± 0.129a	21.855 ± 0.178b
*Eggplant*	Black net	37.336 ± 0.075b	45.848 ± 0.078a	−15.623 ± 0.018c	25.464 ± 0.693a
	Blue net	36.352 ± 0.087c	43.879 ± 0.122c	−14.301 ± 0.122b	22.203 ± 0.064b

	Significance	***	***	***	**

Different letters within columns indicate significant mean differences according to Tukey’s HSD test (p = 0.05). ns, **, and*** denote non-significant or significant effects at p ≤ 0.01 and 0.001, respectively. Data are mean values ± standard error, n = 3.

### Effects of Light Intensity and Quality on Mineral and Pigment Accumulation in Seedlings

The change in intensity and quality of light affects the hormonal pathways of signal molecules involved in transmitting light signals to the roots, which regulates the uptake of nutrients in seedlings (Turnbull, 2011). Except for tomato seedlings, the unshading condition reduced nitrate (on average, –60.27, –20.82, –34.26%, in zucchini squash, watermelon, and eggplant, respectively) compared to shadings conditions (Table 4). Under unshading conditions, the demand for sugars and organic nitrogen is high (higher photosynthetic activity), and vacuolar nitrate is exchanged for soluble sugars and organic acids. Moreover, under shading conditions, nitrate may be a readily available vacuolar osmoticum (Poorter et al., 2019). This could explain the reduction of nitrate in our study under unshaded conditions. Compared to the No shading treatment, the blue net reduced nitrate by 18.57% in tomato seedlings. Similarly, Ohashi-Kaneko et al. (2007) and Li et al. (2019) reported nitrate reduction in plants exposed to blue light. A similar trend was observed in zucchini squash and eggplant seedlings (Table 4). Nitrate performs critical physiological and biochemical functions in adult plants (Hasanuzzaman et al., 2018). However, there is a lack of references and contributions explaining the importance of nitrate in vegetable seedlings in the literature. On the contrary, many authors have investigated the importance of nutritional status in forest seedlings, finding a link between nutritional status and survival in the field. Professional nursery growers use different cultural practices to harden container-grown seedlings, such as reducing day length and temperature but, most importantly, changing the fertilization regime to high-quality stock seedlings (Grossnickle, 2012). Proper fertilization in the nursery can affect the survival of seedlings after planting because they have limited ability to take the necessary nutrients from the soil during the establishment phase. A comparative study of forest seedlings (Van den Driessche, 1991) showed that adequate nursery fertilization increased field survival by about 60%. Similarly, Van den Driessche (1984) and Van den Driessche (1988) observed survival in seedlings of *Pseudotsuga menziesii* (Mirb.) Franco) was related to nitrogen concentration at planting.

**TABLE 4:** Effects of shading and light quality on minerals accumulation of zucchini squash (*C. pepo*), watermelon (*C. lanatus)*, tomato (*S. lycopersicum)*, and eggplant (*S. melongena)* seedlings.

Crop	Treatment	Nitrate	P	K	Ca	Mg
		(mg kg^–1^ fw)	(g kg^–1^ dw)			
	No shading	95.494 ± 0.612c	1.661 ± 0.010b	34.934 ± 0.076a	4.009 ± 0.063a	1.906 ± 0.022b
*Zucchini squash*	Black net	282.574 ± 5.331a	1.759 ± 0.020a	34.391 ± 0.122b	3.741 ± 0.030b	2.039 ± 0.039a
	Blue net	198.165 ± 1.745b	1.762 ± 0.017a	33.899 ± 0.117c	3.645 ± 0.022b	1.952 ± 0.007ab
	Significance	***	**	***	**	*
	No shading	17.315 ± 0.712b	0.555 ± 0.008b	26.681 ± 0.206	5.982 ± 0.168c	1.757 ± 0.018c
*Watermelon*	Black net	21.312 ± 0.418a	0.579 ± 0.003ab	26.598 ± 0.117	7.156 ± 0.047b	1.935 ± 0.031b
	Blue net	22.426 ± 0.165a	0.591 ± 0.005a	26.675 ± 0.150	9.268 ± 0.243a	2.168 ± 0.050a
	Significance	***	**	*ns*	***	***
	No shading	81.002 ± 2.047a	0.686 ± 0.005b	16.148 ± 0.363b	3.842 ± 0.133b	1.495 ± 0.032b
*Tomato*	Black net	74.137 ± 2.800ab	0.605 ± 0.004c	16.807 ± 0.217b	6.842 ± 0.042a	1.913 ± 0.012a
	Blue net	65.958 ± 1.625b	0.780 ± 0.023a	18.503 ± 0.121a	6.597 ± 0.040a	1.999 ± 0.030a
	Significance	**	***	**	***	***
	No shading	133.763 ± 8.286c	2.814 ± 0.054a	40.201 ± 0.539a	3.336 ± 0.066a	1.929 ± 0.058b
*Eggplant*	Black net	237.072 ± 9.772a	1.390 ± 0.035c	35.203 ± 0.634b	3.049 ± 0.094ab	2.097 ± 0.065b
	Blue net	169.909 ± 4.223b	2.289 ± 0.007b	35.884 ± 0.386b	2.708 ± 0.106b	2.628 ± 0.045a
	Significance	***	***	***	**	***

Different letters within columns indicate significant mean differences according to Tukey’s HSD test (p = 0.05). ns, *, ** and *** denote non-significant or significant effects at p ≤ 0.05, 0.01, and 0.001, respectively. Data are mean values ± standard error, n = 3.

Phosphorus is an essential macronutrient involved in photosynthesis, energy metabolism, respiration, and maintenance of cellular structures. It drives enzyme activation, stimulates root and stem development, and constitutes ATP and nucleic acids (DNA and RNA) (Malhotra et al., 2018). Xu et al. (2021) reported that phosphorus utilization efficiency increases with high light intensity within a threshold, beyond which adverse effects on nutrient uptake were observed. However, as observed in our study, the species do not have a univocal response (Table 4). In zucchini squash seedlings under unshading treatment, phosphorus decreased by 5.57%, compared to the black net. On the contrary, an opposite trend was observed in tomato and eggplant seedlings (+ 13.39 and + 102.45%, respectively), compared to the black net.

Potassium is the most abundant inorganic cation in plants that performs a wide range of metabolic functions such as osmoregulation and cell homeostasis and takes a role in enzymatic activation and protein synthesis (Amtmann and Rubio, 2012). The No shade treatment significantly increased potassium in eggplant seedlings (on average, + 13.14%) compared to shading treatments, while in tomato seedlings, the highest potassium values were obtained under the blue net (Table 4). Probably, blue radiation directly influenced potassium uptake. In fact, it was reported in the literature that blue light can regulate stomatal opening and, consequently, promote nutrient uptake through transpiration-induced mass flow (Kinoshita et al., 2001; Van Ieperen et al., 2012). Watermelon seedlings did not show significant differences in potassium between treatments, while in zucchini squash seedlings, the highest value (34.934 g kg^–1^ dw) was obtained in the No shading treatment (Table 4).

Like potassium, the highest calcium was obtained in the No shading condition in zucchini squash. In contrast, shade provided the highest calcium values in watermelon and tomato compared to the No shading treatment (Table 4). Under shading treatments, the highest magnesium content in watermelon, tomato, and eggplant seedlings was obtained (Table 4). Specifically, watermelon and eggplant showed an increase in magnesium under the blue net, while there was no difference between shading nets in tomato seedlings. However, it should be noted that the increase in magnesium under shading treatments in tomato and eggplant seedlings was well correlated with the increase in total chlorophyll (Table 5). Mg is the central atom of the chlorophyll a and b porphyrin ring of green plants (Bohn et al., 2004).

**TABLE 5:** Effects of shading and light quality on the accumulation of pigments of zucchini squash (*C. pepo*), watermelon (*C. lanatus)*, tomato (*S. lycopersicum)*, and eggplant (*S. melongena)* seedlings.

Crop	Treatment	Total Chlorophyll	Carotenoids	Chlorophyll a/b
		(mg g^–1^ fw)		
	No shading	1.741 ± 0.022c	0.237 ± 0.006a	1.378 ± 0.030a
*Zucchini squash*	Black net	1.885 ± 0.002b	0.210 ± 0.004b	1.308 ± 0.017a
	Blue net	2.074 ± 0.002a	0.145 ± 0.002c	1.125 ± 0.025b
	Significance	***	***	***
	No shading	1.397 ± 0.009b	0.345 ± 0.001	1.838 ± 0.005
*Watermelon*	Black net	1.359 ± 0.006b	0.346 ± 0.002	1.884 ± 0.045
	Blue net	1.524 ± 0.015a	0.349 ± 0.002	1.774 ± 0.036
	Significance	***	ns	ns
	No shading	1.040 ± 0.026b	0.363 ± 0.002a	1.768 ± 0.032b
*Tomato*	Black net	1.161 ± 0.006a	0.366 ± 0.001a	1.967 ± 0.014a
	Blue net	1.138 ± 0.007a	0.348 ± 0.003b	1.982 ± 0.021a
	Significance	**	**	***
	No shading	1.395 ± 0.004c	0.335 ± 0.003a	1.691 ± 0.020a
*Eggplant*	Black net	1.450 ± 0.011b	0.317 ± 0.003b	1.642 ± 0.003a
	Blue net	1.529 ± 0.017a	0.296 ± 0.005c	1.539 ± 0.007b
	Significance	***	***	***

Different letters within columns indicate significant mean differences according to Tukey’s HSD test (p = 0.05). ns, ** and *** denote non-significant or significant effects at p ≤ 0.01, and 0.001, respectively. Data are mean values ± standard error, n = 3.

Plants are endowed with sophisticated photoreceptors that transduce the light signal. Chlorophylls (a and b) absorb photons in the blue and red regions and drive metabolic processes by “collecting” energy (Carvalho et al., 2011). It is not surprising that changing the intensity and quality of light affected pigment biosynthesis. Regardless of light intensity, the light quality modification (Blue net) increased total chlorophyll in zucchini squash, watermelon and eggplant, compared to the No shading and black net treatments (Table 5). Plants adapt their chlorophyll pigment content to the light spectrum, and our results are in line with previous findings in lettuce (Son et al., 2012) and cucumber (Li et al., 2019). Similarly, Hogewoning et al. (2010) reported an increase in total chlorophyll in cucumber under R/B = 1 ratio, the same as the blue net used in our experiment. In watermelon, the increase in total chlorophyll under the blue net showed the same trend as the SPAD index (Table 3), as previously reported by Son et al. (2012) in lettuce. In tomato seedlings, the highest chlorophyll content was recorded in shading treatments (Table 4). Probably, tomato plants felt the reduction in light intensity (but not quality) and produced more photosynthetic pigments to absorb more light energy. Although chlorophyll content is reported in the literature as positively associated with photosynthetic capacity and indirectly with productivity (Son et al., 2012), our results do not correlate positively with shoot dry weight (Table 1). However, for nursery seedlings, this result could positively correlate with survival during plant establishment, in addition to providing a productive boost to adult plants.

Common adaptations to irradiation include an increase in the chlorophyll a/b ratio, a parameter that is proposed as a biological assay to evaluate the light environment (Fritschi and Ray, 2007). However, Table 5 does not show a clear trend among species for this parameter. Under the blue net, in zucchini squash and eggplant seedlings, chlorophyll a/b ratio decreased as total chlorophyll increased, attributable to an increased chlorophyll b production under blue light (data not shown). In contrast, we observed an increase in chlorophyll a/b in tomato seedlings grown under shading compared to the No shading condition. Watermelon did not show significant differences in chlorophyll a/b or carotenoid for both intensity and quality of light.

Carotenoids are accessory pigments that capture light and transfer energy to chlorophylls and have photoprotective and antioxidant functions (Carvalho et al., 2011). In zucchini squash and eggplant seedlings, carotenoids increased as the light intensity increased (on average, + 33.52 and + 9.48%, respectively), compared to shading conditions. Our results reflect the role of carotenoids in protecting the leaves from excessive light. Carotenoids probably protected the photosynthetic machinery from high light intensity under No shading treatment (Dietz, 2015). Not least, in tomato seedlings, the blue net reduced carotenoids by 4.92 and 4.13%, compared to the black net and the No shading treatments, respectively.

### Cluster Heatmap of the Effects of Light Intensity and Quality on Morphometric and Quality Indices, Minerals, Colorimetric Parameters, and Pigments Accumulation in Seedlings

Heat maps were made to provide a detailed view of the seedlings’ morphometric, quality, mineral, color, and pigment parameters under different light treatments (light intensity and quality). In general, a different response was observed between families (*Solanaceae* and *Cucurbitaceae*) and between species.

**FIGURE 2:**
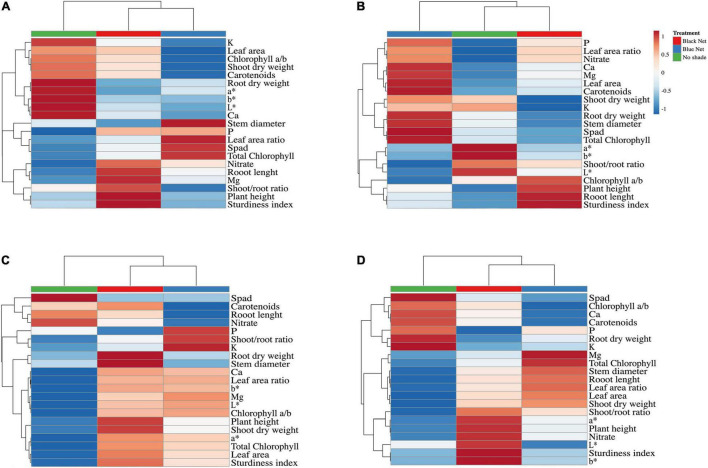
Heatmap analysis summarizing the results of morphometric and quality indices, minerals, colorimetric parameters, and accumulation of pigments of zucchini squash (*C. pepo*) **(A)**, watermelon (*C. lanatus*) **(B)**, tomato (*S. lycopersicum*) **(C)**, and eggplant (*S. melongena*) **(D)** seedlings. Original values are ln(x + 1)-transformed. Columns with similar annotations are collapsed by taking the mean inside each group. The rows are centered; unit variance scaling is applied to the rows. Both rows and columns are clustered using Euclidean distance and complete linkage.

Except for watermelon (Figure 2B), heatmaps analyses of aggregate data in zucchini squash (Figure 2A), tomato (Figure 2C), and eggplant (Figure 2D) identified two main clusters corresponding to the high light intensity treatment (No shading) and shading treatments (Black net and Blue net) (Figure 2). Two separate sub-clusters (Black net and Blue net) were defined under the second cluster indicating that shading was the main clustering factor, while light spectrum modification was the second.

In zucchini squash, blue net reduced leaf area, chlorophyll a/b ratio, shoot and root dry weight, shoot/root ratio, sturdiness index, increased stem diameter, specific leaf area, and total chlorophyll content (Figure 2A). Similarly, the blue net increased the stem diameter, total chlorophyll content, calcium and magnesium concentrations, and carotenoids in watermelon while reducing the shoot/root ratio and the chlorophyll a/b ratio (Figure 2B). In contrast to the findings of *Cucurbitaceae*, in tomato and eggplant seedlings, the blue net had less effect on size reduction (Figure 2). In tomato seedlings, an increase in stem diameter was observed under a black shading net, leading at the same time to the rise in height and thus to a higher sturdiness index (Figure 2C). In tomato seedlings, unshading conditions resulted in lower leaf area, lower chlorophyll a/b ratio, lower plant height, lower shoot/root ratio and root dry weight, lower sturdiness index, lower specific leaf area, lower total chlorophyll content (Figure 2C). In eggplant seedlings, the black net increased plant height and high sturdiness index. High light intensity reduced the shoot/root ratio and shoot dry weight, leaf area, specific leaf area, root length, total chlorophyll, and stem diameter while increasing the chlorophyll a/b ratio (Figure 2D).

### Effects of Light Intensity and Quality on the Metabolic Profile of Seedlings

**FIGURE 3:**
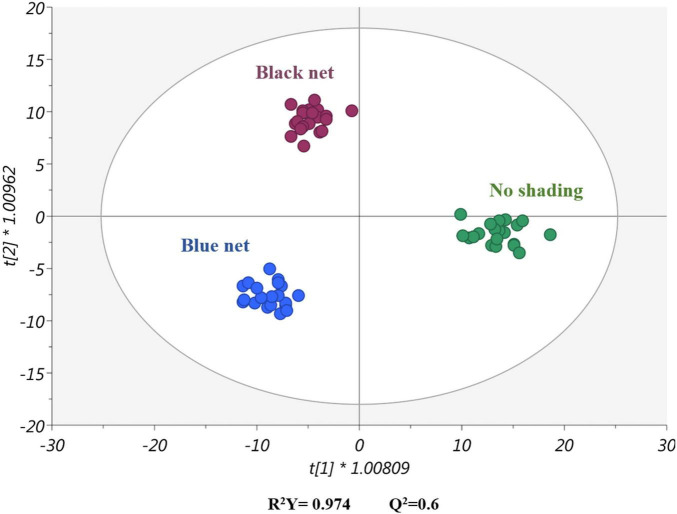
A score plot of orthogonal projection to latent structures discriminant analysis (OPLS-DA) supervised modeling carried out on untargeted metabolomics profiles of zucchini squash (*C. pepo*), watermelon (*C. lanatus*), tomato (*S. lycopersicum*), and eggplant (*S. melongena*) leaves and considering the light quality and intensity as a factor.

The metabolic profiles of *Cucurbitaceae* (zucchini and watermelon) and *Solanaceae* (tomato and eggplant) seedlings were obtained by using an untargeted metabolomics approach to better understand the effect of shading on the physiological process. More than 4,000 metabolites were detected through UHPLC-QTOF-MS analysis, and a comprehensive list of annotated compounds is reported in the Supplementary Table 1. To identify a general trend in plant response to light modulation, only the shading conditions were considered as a factor for supervised multivariate statistics, and all the species were investigated together for the metabolomics analysis. The entire dataset was analyzed using the supervised OPLS-DA, resulting in a clear separation of samples in the score plot based on the net shading (Figure 3). In fact, the first latent vector t[1] clearly indicated that shading triggered a specific metabolic signature different from the unshading plants. Moreover, the second latent vector t[2] showed that shading plants presented a distinctive metabolic profile depending on the net (blue or black).

Therefore, as suggested by the morphometric and quality indices of seedlings, the metabolic profiles indicated a precise modulation of the leaf at the molecular level when changing light quality and intensity, which corroborates morphological changes. In this sense, Wang P. et al., 2020 reported the modulation of the biochemical fingerprint of tea plants under different light intensities, in particular under three supplemental intensities of blue light.

**FIGURE 4:**
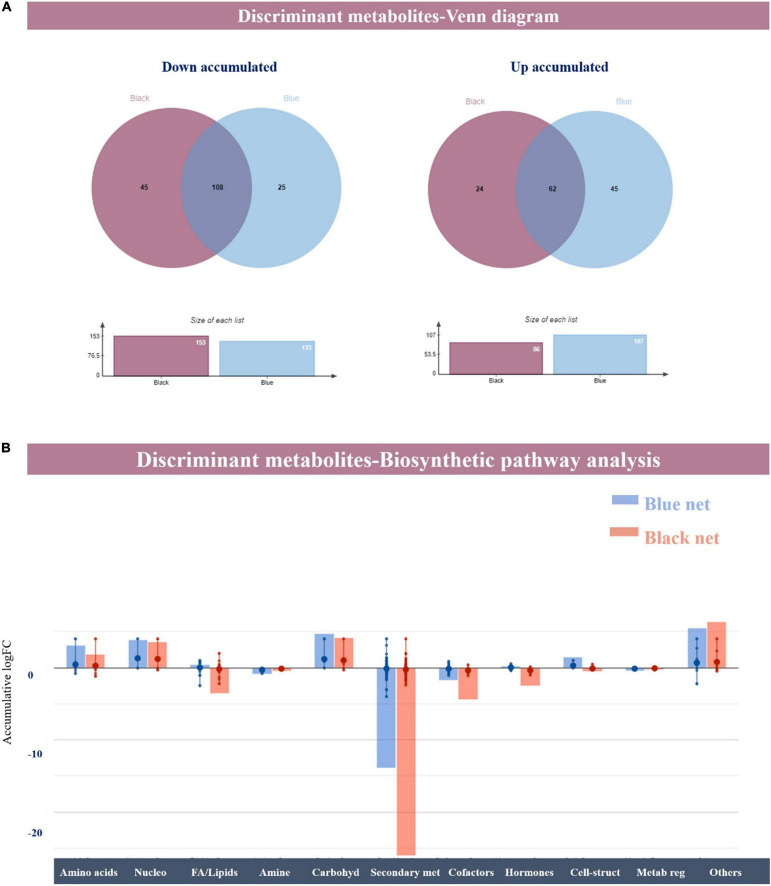
**(A)** A Venn diagram summarizing the discriminant metabolites down and up accumulated under blue and black nets compared to the unshading plants, as resulted from the variable importance in projection (VIP) analysis (VIP score 1.3). **(B)** Metabolic processes are impaired by shading (blue and black net). Metabolites resulted as discriminant from the VIP analysis, and their fold-change values were elaborated using the Omic Viewer Dashboard of the PlantCyc Pathway Tool software (www.pmn.plantcyc.com). The large dots represent the average (mean) of all log Fold-change (FC) for metabolites, and the small dots represent the individual log FC for each metabolite. The x-axis represents each set of subcategories, while the y-axis corresponds to the cumulative log FC. Nucleo: nucleosides and nucleotides; FA/Lipids: fatty acids and lipids; Amines: amines and polyamines; Carbohyd: carbohydrates; Secondary met: secondary metabolism; Cofactors: cofactors, prosthetic groups, electron carriers, and vitamins; Cell-structures: plant cell structures; Metab reg: metabolic regulators.

Once confirmed that shading strongly modulated the leaf metabolic profile regardless of the plant species, the discriminant metabolites that explain the separation of profiles in the score plot were selected by the variable importance in projection (VIP) analysis. In particular, the compound having a VIP score > 1.3 was retained for further investigation (Supplementary Table 2). Venn diagrams show that most compounds overlap for the black and blue net, indicating a shared effect of shading (Figure 4A) according to previous studies that pointed out light quality and intensity as essential factors in plant metabolism (Li et al., 2020; Wang P. et al., 2020). However, 45 and 24 compounds were down and up accumulated, respectively, in the sole presence of the black net. In comparison, 25 and 45 compounds were down and up accumulated, respectively, exclusively in the presence of the blue net. Regardless of the specific metabolites, both black and blue net presented a high ratio of down/up accumulated compounds since the black net decreased the biosynthesis of 153 compounds while increasing the biosynthesis of 86 while for the blue net 133 compounds decreased and 107 increased. Looking at the specific metabolites, the most discriminant markers were those related to terpenes and phenylpropanoids possessing the highest VIP score and indicating their strong implication in plant response to light intensity and quality, as previously reported (Wang P. et al., 2020). Nevertheless, several classes of metabolites including primary and secondary metabolism were found to be discriminant in plant response. The influence of blue light quality and intensity on plant metabolism has been previously confirmed through the metabolomic and transcriptomic approaches that revealed that low-intensity blue light, medium-intensity blue light, and high-intensity blue light triggered a reprogramming in essential physiological processes and secondary metabolism (Wang P. et al., 2020). Moreover, it has been reported that shading alters nitrogen and carbon metabolism, which explains the changes observed at the biochemical level and is supported by the nitrate concentration data under shading (Li et al., 2020; Table 4).

Considering the chemical diversity of VIP compounds, these 238 metabolites were further analyzed by classifying them into the plant biosynthetic pathways (Figure 4B). Figure 4B depicts the biochemical reprogramming triggered by light intensity and quality in plant leaves regardless of the species. Overall, shading seemed to positively modulate those pathways related to primary metabolism (i.e., amino acids, nucleotides, and carbohydrate biosynthesis) while compromising secondary metabolism. However, those molecules involved in several essential processes as phosphoenolpyruvate or cabamoyl-aspartate increased under shading while citrate and isocitrate decreased as a common response. Previously studies reported that energy metabolism was affected by shading. In particular, Li et al. (2020) observed a decrease in sugar content and suggested a lower need for energy under shading conditions that lead to changes in carbon flux from the synthesis of glucose to a feedback mechanism by shifting stored glucose to amino acid metabolism instead of normal carbon metabolism.

On the one hand, according to our results, Lakshmanan et al. (2015) observed an increase in the flux of metabolic pathways after blue light treatment in *Arabidopsis thaliana*, including the biosynthesis of lipids. Our findings revealed that fatty acids and lipid biosynthesis were positively regulated by the blue net rather than the black net. In agreement with our results, Wang P. et al., 2020 observed that blue light promoted lipid biosynthesis, mainly sterols and sphingolipids that are membrane structural components and might act as signal molecules. Notably, compounds classified into “cofactors, carriers, and vitamin biosynthesis” were modulated by black and blue shading. 6-methoxy-3-methyl-2-all-*trans-*decaprenyl-1,4-benzoquinol, 3-demethylubiquinol-9, 3-demethylubiquinol-9, and 3-non-aprenyl-4-hydroxybenzoate decreased under shading while 3,4-dihydroxy-5-all-*trans-*decaprenylbenzoate increased, pointing out the modulation of the ubiquinone pathway and respiratory electron transport in this response. In contrast, thiamine and thiamine diphosphate were positively regulated by the blue net and negatively modulated by the black net. Moreover, several compounds related to the biosynthesis of chlorophylls upstream (i.e., Mg-protoporphyrin, haematoporphyrin, uroporphyrin) were positively modulated under shading, according to the physiological measures including the uptake of Mg, while chlorophyll degradation products (protochlorophyll a) decreased (Bohn et al., 2004; Hernández and Kubota, 2016).

On the other hand, secondary metabolism biosynthesis was strongly repressed by both the blue and black nets. This repression is reflected in the marked down accumulation of nitrogen-containing compounds, which were the most affected class of secondary metabolites. This might be explained by the modulation in amino acid metabolism, phenylpropanoids, and terpenes being more marked for black shading (Li et al., 2020). Despite this, blue net provokes an accumulation of precursors of N-containing metabolites and the accumulation of some phenylpropanoids [dalnigrein 7-*O*-β-D-apiofuranosyl-(1–6)-β-D-glucopyranoside, amorphigenin, cyanidin 3-*O*-(6″-*O*-malonyl)-β-glucoside, 4-hydroxycoumarin] according to previous results. In fact, light intensity and shading regulate the expression of the genes and the activity of enzymes involved in the biosynthesis of flavonoids, anthocyanin, catechins, and flavanols (Li et al., 2020). In particular, blue light not only affects the synthesis of flavonoids, even if this modulation (positive or negative) depends on plant species, but also the light intensity (Wang P. et al., 2020). In addition, flavonoid metabolism is also influenced by the TCA cycle and the biosynthesis of carbohydrates and amino acids, indicating a complex network between primary and secondary metabolism under shading rather than a direct effect on the specific expression of key genes (Li et al., 2020). In contrast, terpenoids seemed to be shading-specific modulated and seemed to be particularly altered by shading, as suggested by the VIP analysis. Precursors, such as mevalonate and squalene, and their final products as sterol and carotenoids and terpene hormones as brassinosteroids were specifically modulated, with their effect being stronger under blue shading in agreement with the general modulation of lipids under shading.

## Conclusion

Light drives many vital processes in plants, which show different morphophysiological responses to varying degrees of light intensity and quality as an adaptation. For example, shading increases leaf area and pigment content, while high light intensity increases photosynthetic activity and shoot growth. However, changing light quality also induces adaptive changes in plants. Due to self-shading, blue light depletion in high-density plants reduces seedling quality (less compactness), driving producers to use chemical size regulators. In our study, we demonstrated that the response of plants to changing light intensity and quality is species-specific. Moreover, the untargeted metabolomics approach allowed us to identify a common pattern across species in response to shading. Considering that light controls essential biochemical and physiological processes, our results highlighted that both primary and secondary metabolism, together with the phytohormone profile, were largely affected by shading, resulting in a biochemical modulation much broader than photosynthesis and phytohormone profiles. These common patterns included plant energy metabolism and lipid biosynthesis and included a down accumulation of secondary pathways, particularly regarding phenylpropanoids.

The morphological changes induced by the different shading conditions corroborate the shift in metabolomic signatures we observed, indicating that a set of biological processes are modulated by shading. The comprehension of the mechanisms involved pivotally supports the implementation of photoselective shading in dedicated applications, toward the definition of more resilient crop production. Such information is of general relevance and is even more important in cropping systems under less favorable intense light conditions, where photoselective shading could represent a sustainable approach. The blue photoselective net used in our experiment modified the spectral quality of light at the canopy level, changing the blue and red portions of the light spectrum (and the relative red: blue ratio) and affecting seedling size as well as nutritional, biochemical, and physiological condition. However, a robust genotype-dependent response to light modification is evident, making further studies in other vegetable seedlings necessary to expand knowledge of the effects of blue photoselective nets.

## Data Availability Statement

The original contributions presented in the study are included in the article/Supplementary Material, further inquiries can be directed to the corresponding author/s.

## Author Contributions

LF, SDP, and YR: conceptualization and project administration. LF, BM-M, MC, LZ, SDP, LL, and YR: methodology, validation, formal analysis, investigation, writing—original draft preparation, and writing—review and editing. LF, BM-M, MC, and LZ: software and data curation. YR and SDP: resources. YR: visualization and supervision. SDP: funding acquisition. All authors contributed to the article and approved the submitted version.

## Conflict of Interest

The authors declare that the research was conducted in the absence of any commercial or financial relationships that could be construed as a potential conflict of interest.

## Publisher’s Note

All claims expressed in this article are solely those of the authors and do not necessarily represent those of their affiliated organizations, or those of the publisher, the editors and the reviewers. Any product that may be evaluated in this article, or claim that may be made by its manufacturer, is not guaranteed or endorsed by the publisher.

## Footnotes

1 https://biit.cs.ut.ee/clustvis/; accessed January 3, 2022.

## Supplementary Material

Supplementary Figure 1 Effects of shading nets on photosynthetically active radiation (PAR). Data are mean values ± standard error, *n* = 3. All mean effects were subjected to a one-way ANOVA analysis. Statistical significance was determined with Tukey’s HSD test at the *p* = 0.05 level.

Supplementary Figure 2 Red:blue ratios of plastic film (No shade), black shading net (Black net), and blue photoselective shading net (Blue net).

Supplementary Figure 3 Complete light spectrum under each net at the surface level of the plants.

Supplementary Table 1 Metabolic profile of *Cucurbitaceae* (zucchini and watermelon) and *Solanaceae* (tomato and eggplant) seedling.

Supplementary Table 2 List of compounds with a variable importance in projection (VIP) score > 1.3.
