# Supplementary figures and images for: Between Light and Shading: Morphological, Biochemical, and Metabolomics Insights Into the Influence of Blue Photoselective Shading on Vegetable Seedlings
Source: Front Plant Sci. 2022 May 25;13:890830. doi: 10.3389/fpls.2022.890830 (PMC9174935; doi:10.3389/fpls.2022.890830)

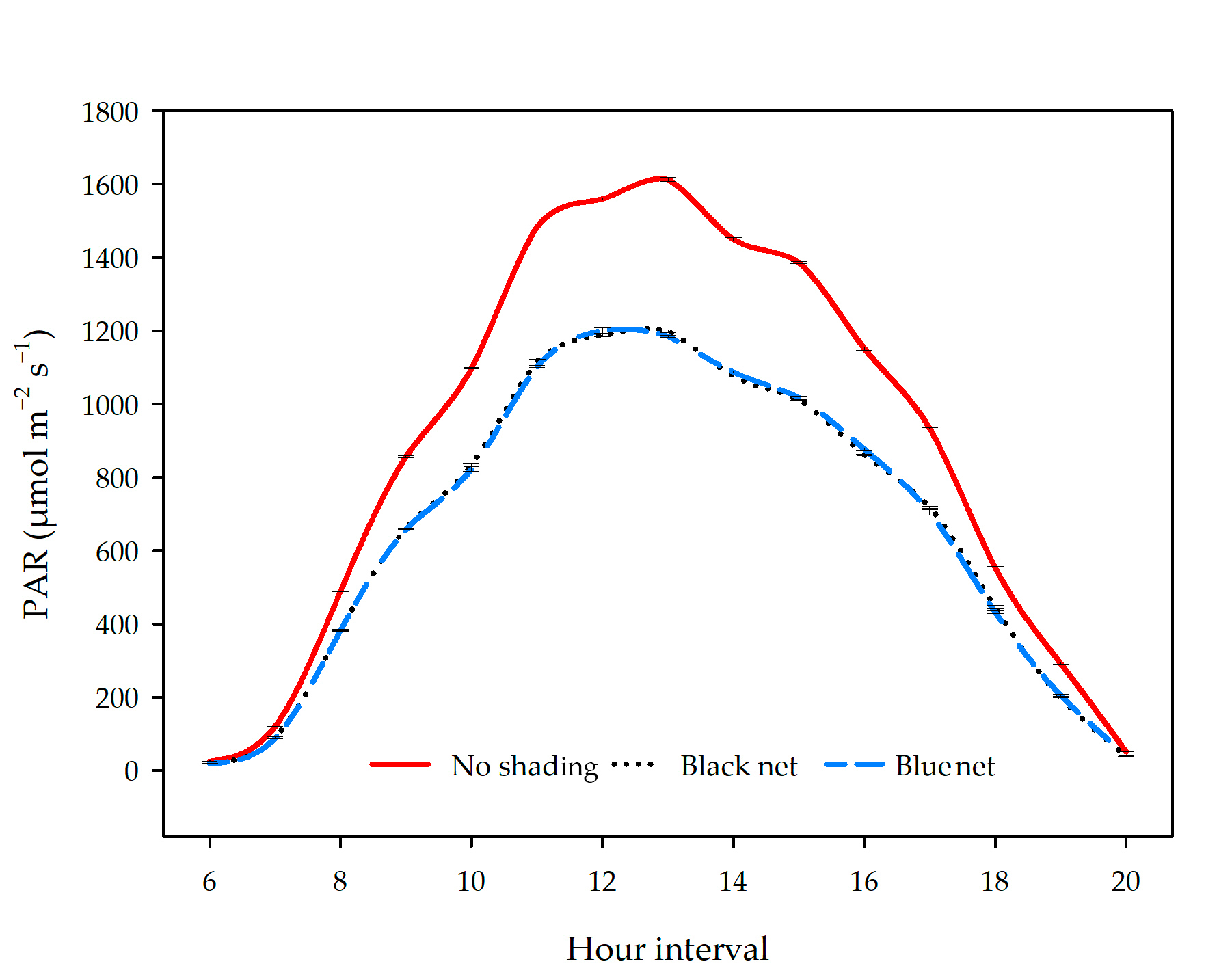

Supplement: Supplementary Figure 1 — Effects of shading nets on photosynthetically active radiation (PAR). Data are mean values ± standard error, n = 3. All mean effects were subjected to a one-way ANOVA analysis. Statistical significance was determined with Tukey’s HSD test at the p = 0.05 level. [file Image_1.JPEG]

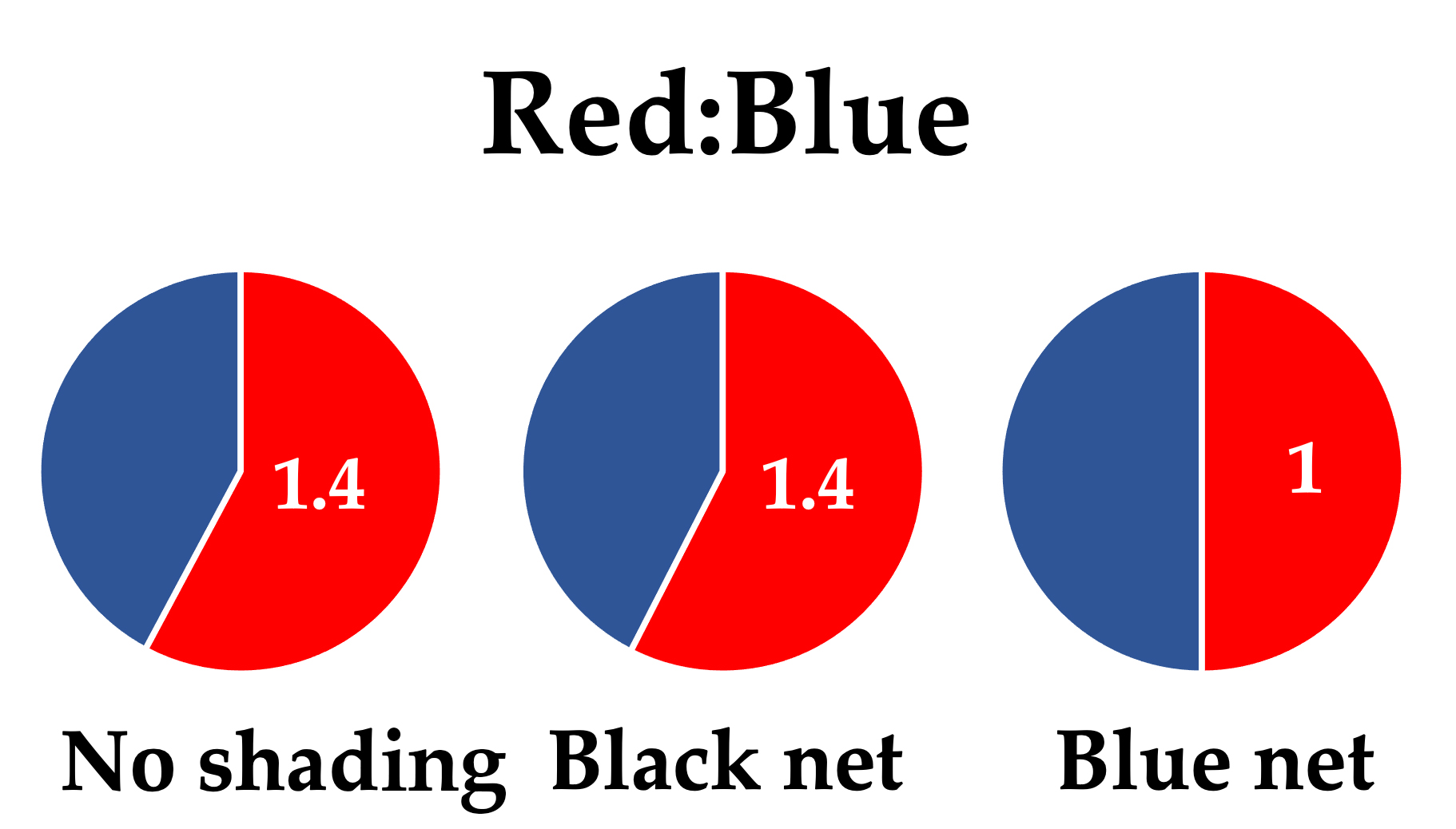

Supplement: Supplementary Figure 2 — Red:blue ratios of plastic film (No shade), black shading net (Black net), and blue photoselective shading net (Blue net). [file Image_2.JPEG]

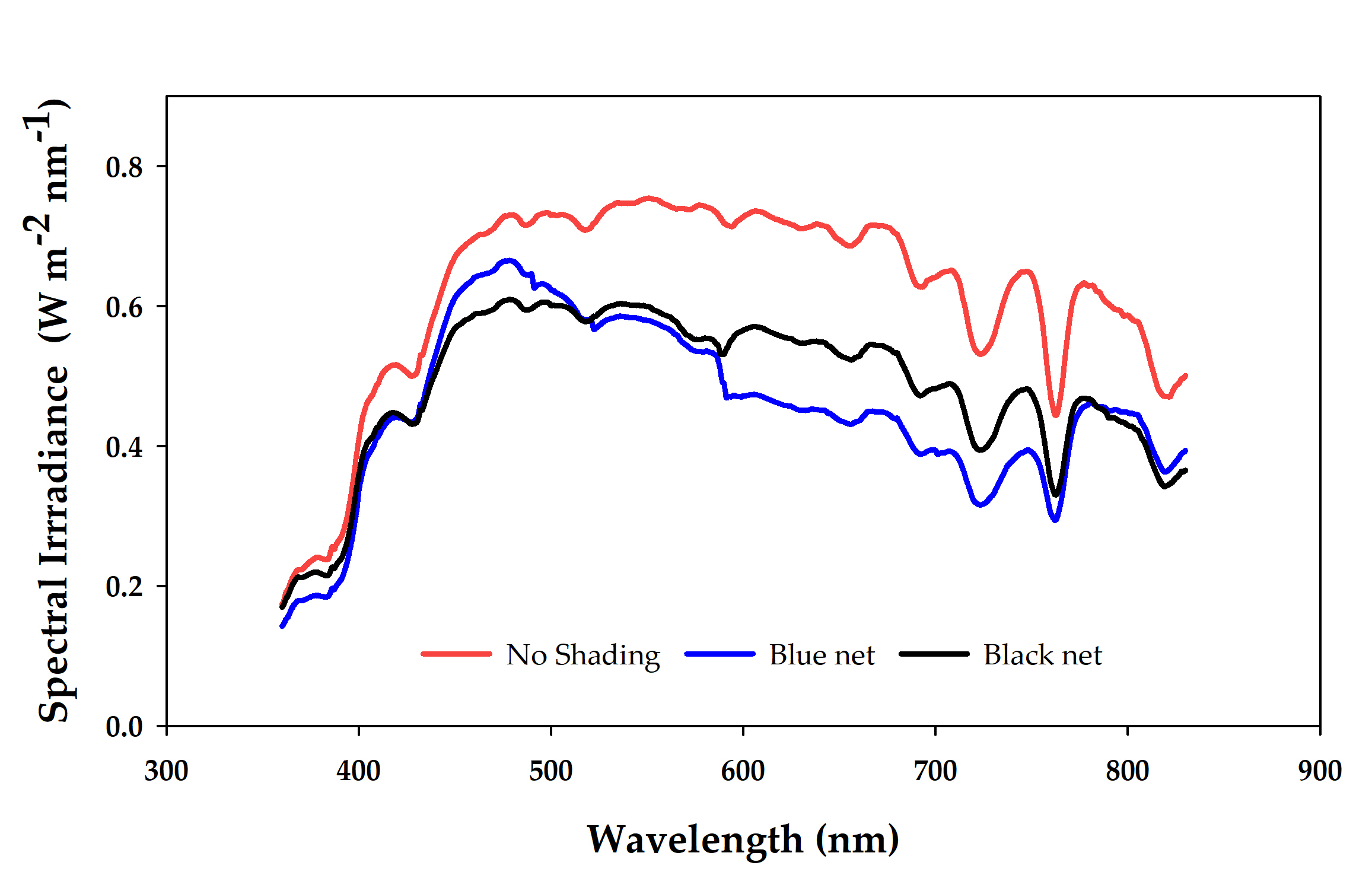

Supplement: Supplementary Figure 3 — Complete light spectrum under each net at the surface level of the plants. [file Image_3.JPEG]
